# Downregulated ADARB1 Facilitates Cell Proliferation, Invasion and has Effect on the Immune Regulation in Ovarian Cancer

**DOI:** 10.3389/fbioe.2021.792911

**Published:** 2021-12-23

**Authors:** Wei Zhu, Zhijie Xu, Meiyuan Huang, Xiang Wang, Xinxin Ren, Yuan Cai, Bi Peng, Qiuju Liang, Xi Chen, Yuanliang Yan

**Affiliations:** ^1^ Department of Pathology, Xiangya Hospital, Central South University, Changsha, China; ^2^ National Clinical Research Center for Geriatric Disorders, Xiangya Hospital, Central South University, Changsha, China; ^3^ Department of Pathology, The Affiliated Zhuzhou Hospital Xiangya Medical College, Central South University, Zhuzhou, China; ^4^ Department of Pharmacy, Xiangya Hospital, Central South University, Changsha, China; ^5^ Key Laboratory of Molecular Radiation Oncology of Hunan Province, Xiangya Hospital, Central South University, Changsha, China

**Keywords:** ADARB1, ovarian cancer, Akt, cell growth, immune regulation

## Abstract

Ovarian cancer (OC) is typically diagnosed at an advanced stage and poses a significant challenge to treatment and recovery. Rencently, Adenosine deaminase RNA-specific B1 (ADARB1), an adenosine-to-inosine (A-to-I) RNA-editing enzyme, has been found to play an essential role in the development of cancer. However, the specific function of ADARB1 in ovarian cancer is still not fully understood. Here, we investigated the effects of ADARB1 on OC biology. By conducting bioinformatics analyses of several public databases, we found significantly decreased ADARB1 expression in OC cells and tissues. Moreover, RT-PCR and western blot showed lower ADARB1 expression in OVCAR3, HO8910pm and A2780 OC cells compared to human normal ovarian epithelial cell IOSE. Cell proliferation assay and clone formation assay showed that overexpression of ADARB1 (ADARB1-OE) inhibited the proliferation of tumor cells. Wound healing and transwell assay indicated that ADARB1-OE could suppress OC cell invasion and metastasis. Kaplan-Meier methods revealed that the patients with low level of ADARB1 displayed poor prognosis. TISIDB databases were further used to analyze the roles of ADARB1 in tumor-immune system interactions in OC patients. Furthermore, ADARB1-OE down-regulated the expression of phosphorylated AKT. Combination of ADARB1-OE and AKT inhibitor MK2206 exerted stronger cell growth inhibition. Thus, our investigation demonstrated that low levels of ADARB1 might be a potential target in the tumorigenesis and prognostic evaluation of OC patients.

## Introduction

Ovarian cancer (OC) is one of the most common gynecological tumors, and the onset age is mainly concentrated in people between 40 and 60 years old, especially in people around 50 years old. It accounts for 2.4–6.5% of the common female malignant tumors, and ranks third in female reproductive system malignant tumors, next only to cervical cancer and endometrial cancer (Brunty et al., 2020; Hidayat et al., 2020). The standard treatment for OC patients is surgery and chemotherapy. Due to the high recurrence rate and therapeutic resistance, the 5-year survival rate for OC patients is about 47% (Demircan et al., 2020; Moschetta et al., 2020). Therefore, it is necessary to search for novel target molecules for improving the treatment and prognosis of OC.

Adenosine deaminase RNA specific B1 (ADARB1) catalyzes the conversion of adenosine (A) to inosine (I) in double-stranded RNAs (Ma et al., 2020). Recently, emerging researches have revealed the relationship between ADARB1 and cancer. In glioma, aberrant expression of ADARB1 can affect the proliferation and invasion of glioma cells by regulating microRNAs (Cesarini et al., 2018) or CDC14B-Skp2 signaling axis (Galeano et al., 2013). The down-regulation of ADARB1 may be related to the pathogenesis of non-small cell lung cancer (Wang et al., 2019; Wang et al., 2020), and low levels of ADARB1 in lung cancer are correlated with shorter first progression (FP), overall survival time (OS) and post-progression survival time (PPS). Moreover, ADAR2 suppresses tumor growth and induces apoptosis by editing and stabilizing IGFBP7 in esophageal squamous cell carcinoma (Chen et al., 2017). However, the association between ADARB1 and OC has not been investigated.

The purpose of our study was to evaluate the role and mechanism of ADARB1 in human OC. Through bioinformatics data analysis and *in vitro* experiments, we found that ADARB1 was downregulated in OC tissues and cell lines, and was correlated with poor prognoses of OC patients. Furthermore, overexpression of ADARB1 (ADARB1-OE) significantly inhibited the proliferation, invasion and metastasis of OC cells by down-regulating AKT phosphorylation.

## Materials and Methods

### Data Acquisition and Reanalysis

As mentioned previously (Yan et al., 2018; Zhou et al., 2019) several bioinformatics network resources were used to reanalyze the molecular profiles of ADARB1 in OC patients (Supplementary Table S1). Gene Expression Profiling Interactive Analysis (GEPIA) (Tang et al., 2017) and University of Alabama Cancer Database (UALCAN) (Chandrashekar et al., 2017) were used to identify the expression profiles of ADARB1 in OC tissues. For the prognostic analysis, the Kaplan–Meier Plotter (Lánczky et al., 2016) and DRUGSURV database (Amelio et al., 2014) were utilized to describe the relationship between ADARB1 expression and patients’ prognosis.

From the cBioPortal web tool (Gao et al., 2013), the genes coexpressed with ADARB1 in OC were downloaded (Supplementary Table S2). Then, the STRING (Szklarczyk et al., 2017) and Cytoscape software (Reimand et al., 2019) were used to complete the protein–protein interaction (PPI) network of these coexpression genes. Then, we utilized the WebGestalt (Wang et al., 2017) to conduct the GO and KEGG pathway analysis of ADARB1 coexpression genes in OC samples.

### Immunohistochemistry

The ovarian cancer tissues and paired adjacent tissues were obtained from the Xiangya hospital, central south university. The ethical approval number is 20210205. Specimens were deparaffinized in xylene and rehydrated in a series of graded alcohol. Antigen retrieval was completed after heating in citrate buffer. Endogenous peroxidase was blocked using 3% H_2_O_2_ for 20 min. The sections were incubated with the primary antibody against ADARB1, p-AKT, CXCL12 and KDR at 37°C for 1 h. Horseradish peroxidase-conjugated secondary antibody was added and incubated at room temperature for 30 min, and 3,3′-diaminobenzitine (DAB) solution was used for color reaction (ZSGB-BIO, China).

### Cells and Reagents

Human OC cells OVCAR3, HO8910pm, A2780 and normal ovarian epithelial cells IOSE were obtained from department of Pathology, School of Basic Medical Sciences, Central South University. OVCAR3, HO8910pm and IOSE were maintained in Roswell Park Memorial Institute (RPMI)-1640 medium (BI, Israel Beit Haemek Ltd.) with 10% fetal bovine serum (FBS), while A2780 was grown in DMEM (BI, Israel Beit Haemek Ltd.) supplemented with 10% fetal bovine serum FBS at 37°C and 5% CO2. MK2206 was purchased from Selleck Chemicals and dissolved in dimethylsulfoxide (DMSO) (Sigma, United States), The exposed concentrations of MK2206 were 5 mM. Antibodies against ADARB1 and p-AKT were purchased from Proteintech (United States, 22248-1-AP and 66444-1-Ig). Antibodies against GAPDH and CXCL12 were purchased from Abclonal (China, AC002 and A1325), Antibody against KDR was purchased from Affinity (China, AF6281). Antibodies against AKT and p-AKT were from Cell Signaling Technology (United States). The second antibody were purchased from ZSGB-BIO.

### Western Blot

Cells were lysed in RIPA buffer and protein concentrations were determined by a BCA protein assay kit (PP1002, BioTeck, Beijing, China). A total of 30–40 μg of protein was separated by 10% SDS-PAGE and electroblotted onto polvinylidene fluoride membranes (Millipore, Merck, United States) for detection using indicated antibodies. Immunoreactions were detected by an imaging system (Alpha FluorChem FC3).

### RNA Extraction and Reverse Transcription PCR

Total RNA was extracted employing TRIzol reagent (Invitrogen) according to the manufacturter’s instruction and reverse transcribed to cDNA using the PrimeScript™ RT reagent kit (Abclonal, China). The RT-PCR assay was conducted through iTaqTM Universal SYBR green Supermix (Abclonal, China), with GAPDH as the internal control. The forward and reverse primer sequences were used as follows: ADARB1: 5′-CGC​GCC​TTT​GTT​TGT​CAT​GTC-3′ and 5′-GGA​AAC​TGA​ACG​AAA​GAC​CTC​AA-3′; GAPDH: 5′-CAG​CAA​GAG​CAC​AAG​AGG​AA-3′ and 5′-ATG​GTA​CAT​GAC​AAG​GTG​CGG-3′. Relative expression levels were decided using the 2-ΔΔCT method.

### MTT Asaay

The logarithmic growth phase cells were cultured in a 96-well culture plate for 6 days with five replicates per group. MTT assay was performed 48 h after transfection. Add 10 μl MTT solution to each well at the end of each day. After 4 h of continuous culture, the culture medium was discarded, and DMSO (150 μl/well) was added 6 days later, and the medium was shaken for 10 min. The cell proliferation rate was calculated by measuring the optical density (OD) of each well at 490 nm on a Microplate Reader.

### Colony Formation Assay

A total of 1,000 cells were seeded in 6-well plates and incubated at 37°C with 5% CO2 for 10 days. Culture plates were performed in duplicates. After a wash with phosphate buffer saline (PBS), cultures were fixed with methanol for 20 min and stained with crystal violet for 15 min. Colonies were examined and calculated.

### Wound Healing Assay

The cells were inoculated into 6-well plates and cultured in complete medium (37°C, 5% CO2) to at least 95% confluence before wounds were created. To measure the cell migration, a plastic 100 ul pipette tip was used to scrape cells in a monolayer to creating wounds. Then, washed them three times in PBS and incubated with FBS-free RPMI-1640 medium. Subsequently, cells were cultured in either medium for 0, 12, 24 h. At the end of the incubation period, phase-contrast microscopy was employed to photograph the wounded area and migration cells at the wounded area. Finally, the extent to which the wound had closed over 24 h was calculated and expressed as a percentage of the difference between times 0 and 24 h using ImageJ software.

### Transwell Invasion Assays

For invasion assays, Transwells (corning, United States) with 8-μm pore size filters covered with matrigel were inserted into 24-well plates. The cells were serum-starved overnight and then added in the upper chamber (5 × 10^4^ cells per insert) and the complete culture medium supplemented with 10% FBS was used as a chemoattractant in the lower chamber. After 24 h of incubation, non-invading cells that remained on the upper surface of the filter were removed, and the cells that had passed through the filter and attached to the bottom of the membrane were fixed in methanol and stained with 0.2% Crystal violet. Numbers of the invasive cells in seven randomly selected fields from triplicate chambers were counted in each experiment under a phase-contrast microscope.

### Statistical Analysis

Statistical analyses were performed using SPSS 22.0 for Windows. Data were presented as mean ± standard deviation (SD) of at least three independent experiments. Statistical analysis was performed using Student’s t test and one-way ANOVA. The Statistical significance is shown in the figures with *p < 0.05, **p < 0.01, ***p < 0.001 and ****p < 0.0001.

## Results

### ADARB1 Was Downregulated in OC Tissues and Cell Lines

**FIGURE 1:**
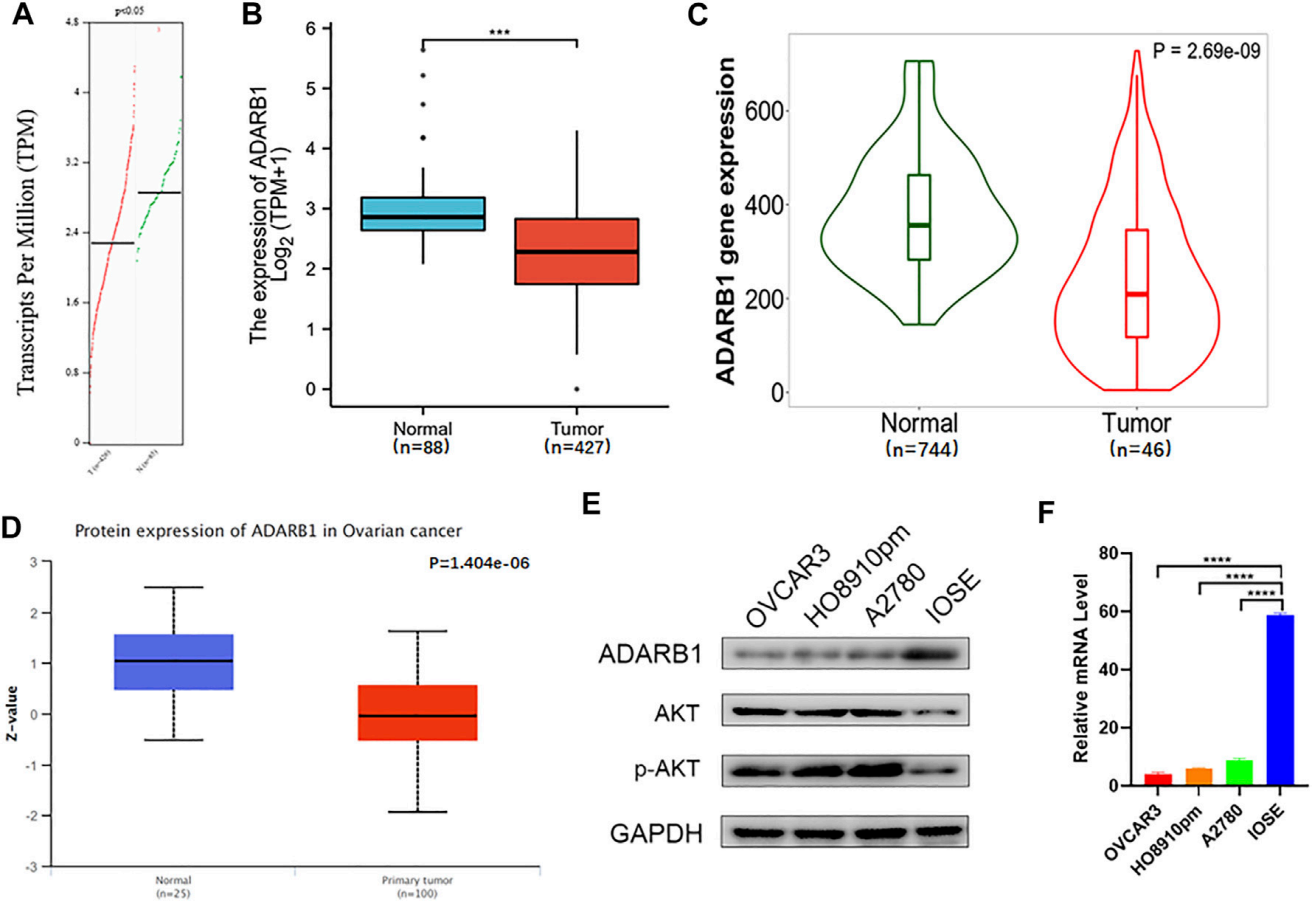
Downregulation of ADARB1 in OC tissues and cell lines. **(A–D)** The expression of ADARB1 was analyzed by the **(A)** GEPIA, **(B)** TCGA, **(C)** TNM plot **(D)** UALCAN databases, respectively. **(E,F)** Expression of ADARB1 was analyzed by RT-PCR and western blot.

**FIGURE 2:**
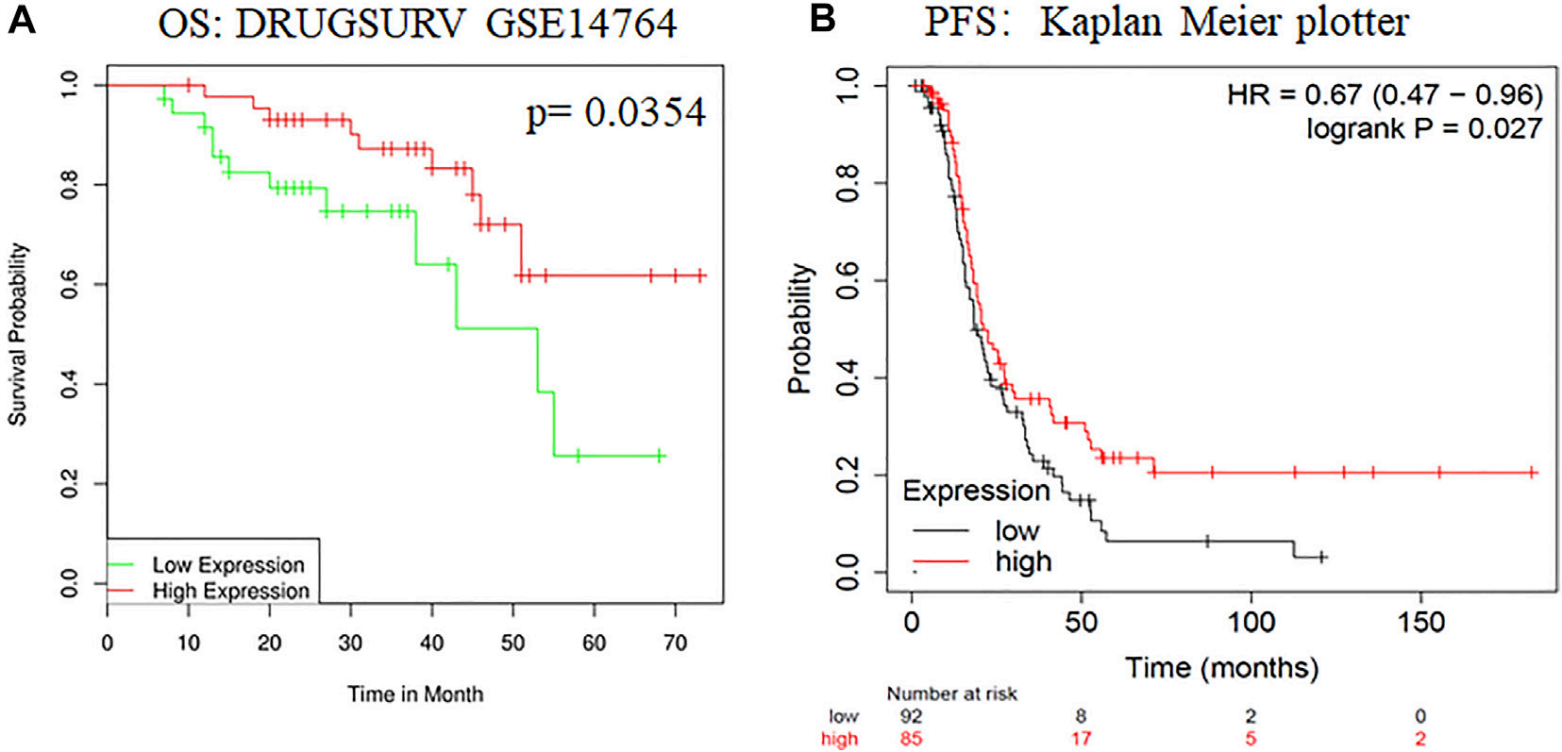
The effects of ADARB1 expression on the prognosis of OC patients. **(A)** The relationship between ADARB1 expression and OS analyzed by DRUGSURV database. **(B)** The relationship between ADARB1 expression and PFS analyzed by Kaplan–Meier Plotter.

To evaluate changes in ADARB1 expression in OC and normal ovarian tissues, we analyzed the expression levels of ADARB1 through several bioinformatics databases. The GEPIA analysis revealed that ADARB1 mRNA expression was significantly decreased in OC tissues (Figure 1A). From TCGA-OV, we discovered the ADARB1 expression was clearly reduced in OC compared with the normal tissues (Figure 1B). Similarly, using the TNM plot (Figure 1C) and UALCAN (Figure 1D), we further confirmed the downregulation of ADARB1 in OC tissues. In addition, we found that OC cells OVCAR3, HO8910pm and A2780 displayed a lower level of ADARB1 compared to the normal ovarian cell IOSE (Figures 1E,F). Moreover, the expression of ADARB1 in tumor tissues was lower than that in adjacent tissues (Supplementary Figure S1). Next, we analyzed the effects of ADARB1 expression on the clinical characteristics of patients with OC. From DRUGSURV database, we found that the patients with low level of ADARB2 displayed poor prognosis in GSE14764 (Figure 2A). In addition, Kaplan-Meier Plotter database was employed to further evaluate the effects of ADARB1 expression on survival, revealing that low levels of ADARB1 expression were correlated with shorter progression-free survival (PFS) (Figure 2B). Therefore, these results suggested that ADARB1 expression levels might serve as an indicator for the clinical prognosis of OC patients. Taken together, the decreased expression of ADARB1 in OC tissues and cell lines suggested its anti-oncogenic roles.

### ADARB1 Could Suppress the Malignant Biological Behaviors of OC Cells

**FIGURE 3:**
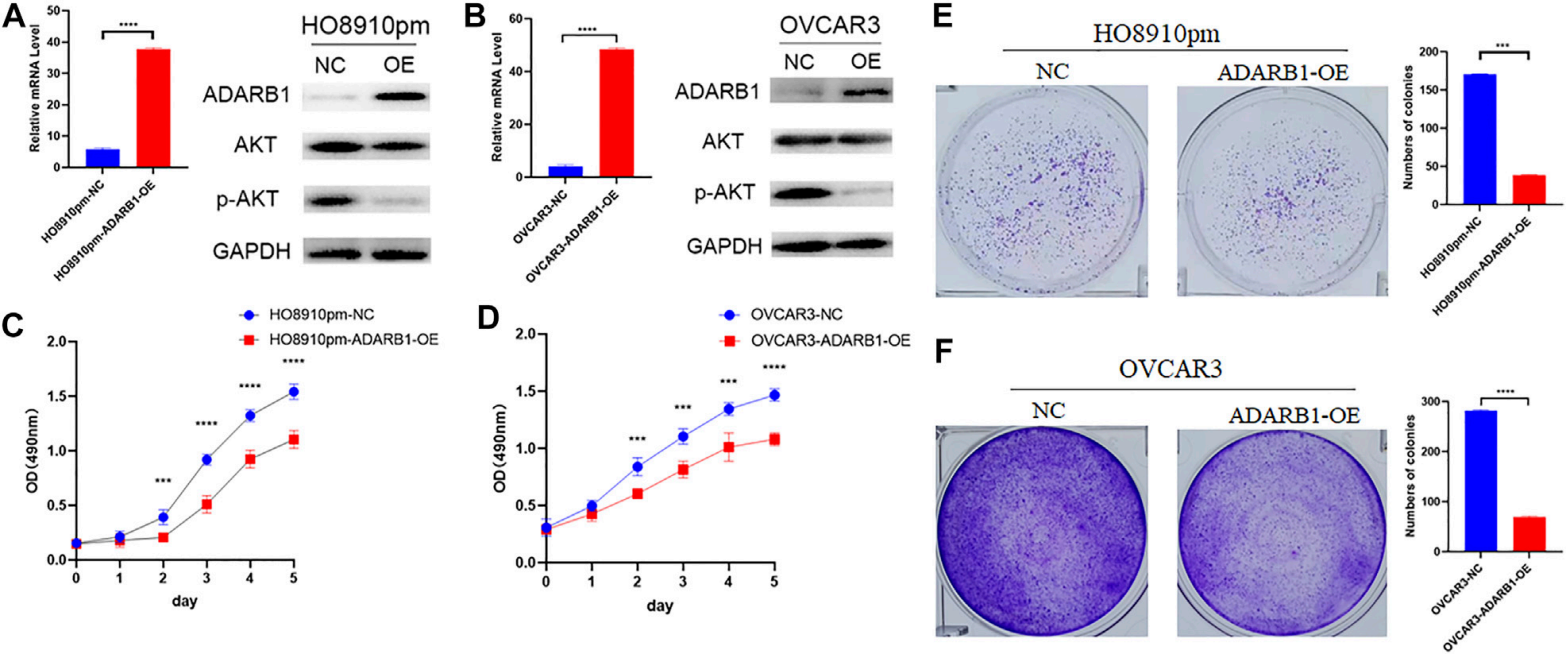
Overexpession of ADARB1 suppressed the proliferation ability of OC cells. **(A,B)** The mRNA levels of ADARB1 after transfected with ADARB1-OE in HO8910pm and OVCAR3 cells. **(C,D)** The results of MTT assay after transfected with ADARB1-OE in HO8910pm and OVCAR3 cells. **(E,F)** The results of colony formation assay after transfected with ADARB1-OE in HO8910pm and OVCAR3 cells.

**FIGURE 4:**
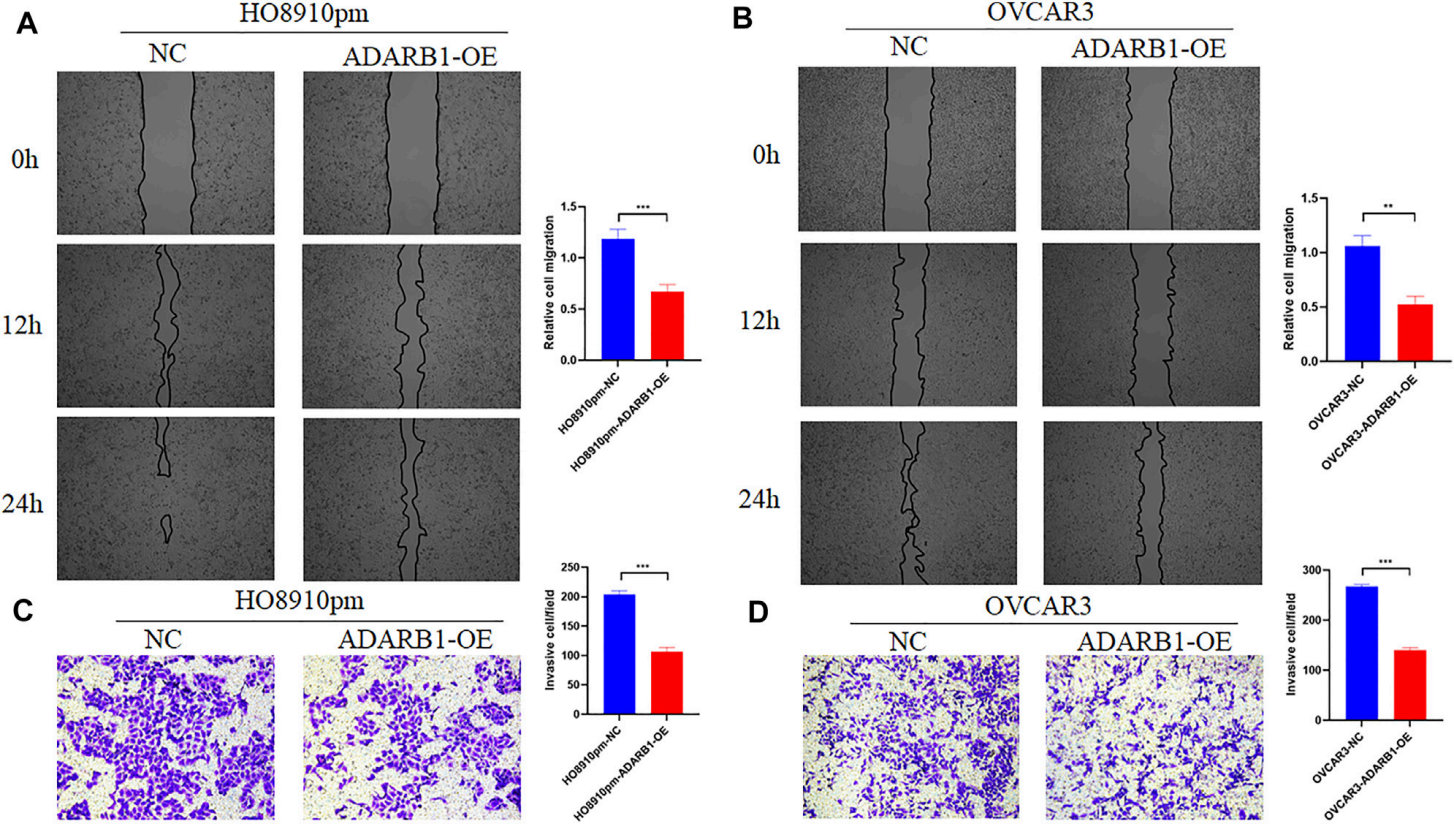
Overexpession of ADARB1 inhibited the migration and invasion ability of OC cells. **(A,B)** The results of wound healing assay after transfected with ADARB1-OE in HO8910pm and OVCAR3 cells. **(C,D)** The results of transwell assay after transfected with ADARB1-OE in HO8910pm and OVCAR3 cells.

To further identify the function of ADARB1 in OC, we overexpressed of ADARB1 in HO8910pm and OVCAR3 ovarian cancer cells (Figures 3A,B). After then, we tested the roles of ADARB1 overexpression in cell proliferation, migration and invasion. The MTT assay and colony formation assay showed that ADARB1 overexpressed significantly decreased the proliferation ability in HO8910pm and OVCAR3 cells (Figures 3C–F). Similarly, we observed that ADARB1-OE inhibited the metastasis and invasion ability in HO8910pm and OVCAR3 cells, as indicated by the wound healing and transwell assays (Figures 4A–D). These results suggest that ADARB1 could suppress the malignant biological behaviors of OC cells.

### ADARB1 Suppressed OC Proliferation and Metastasis by Inhibiting AKT Phosphorylation

**FIGURE 5:**
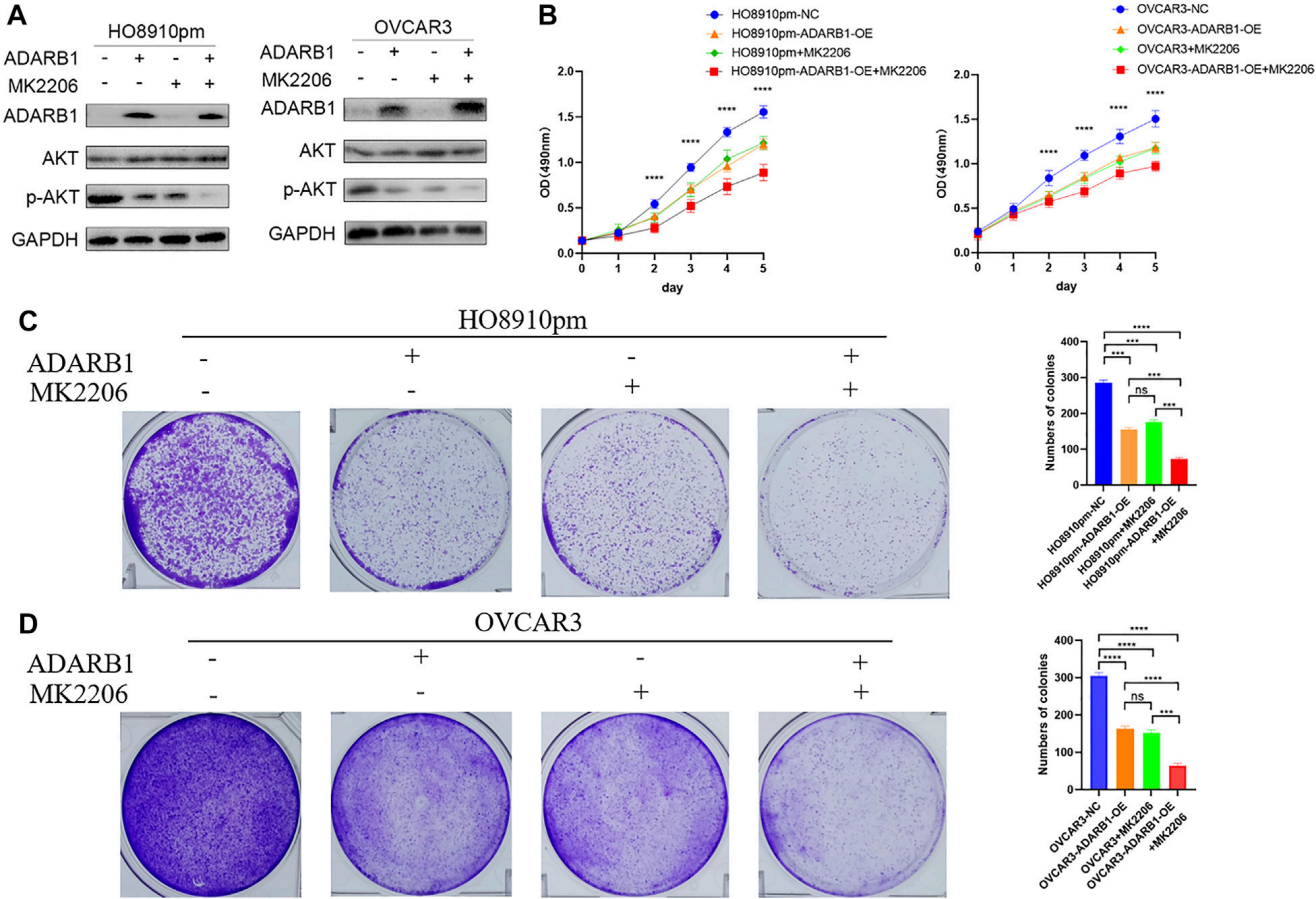
AKT pathway was involved in ADARB1-reduced cell proliferation. **(A)** HO8910pm and OVCAR3 cells treated with ADARB1-OE and/or AKT inhibitors MK2206 subjected to Western blot assays. **(B)** The MTT assay were performed after HO8910pm and OVCAR3 cells treated with ADARB1-OE and/or AKT inhibitors MK2206. **(C,D)** The colony formation assay were performed after HO8910pm and OVCAR3 cells treated with ADARB1-OE and/or AKT inhibitors MK2206.

**FIGURE 6:**
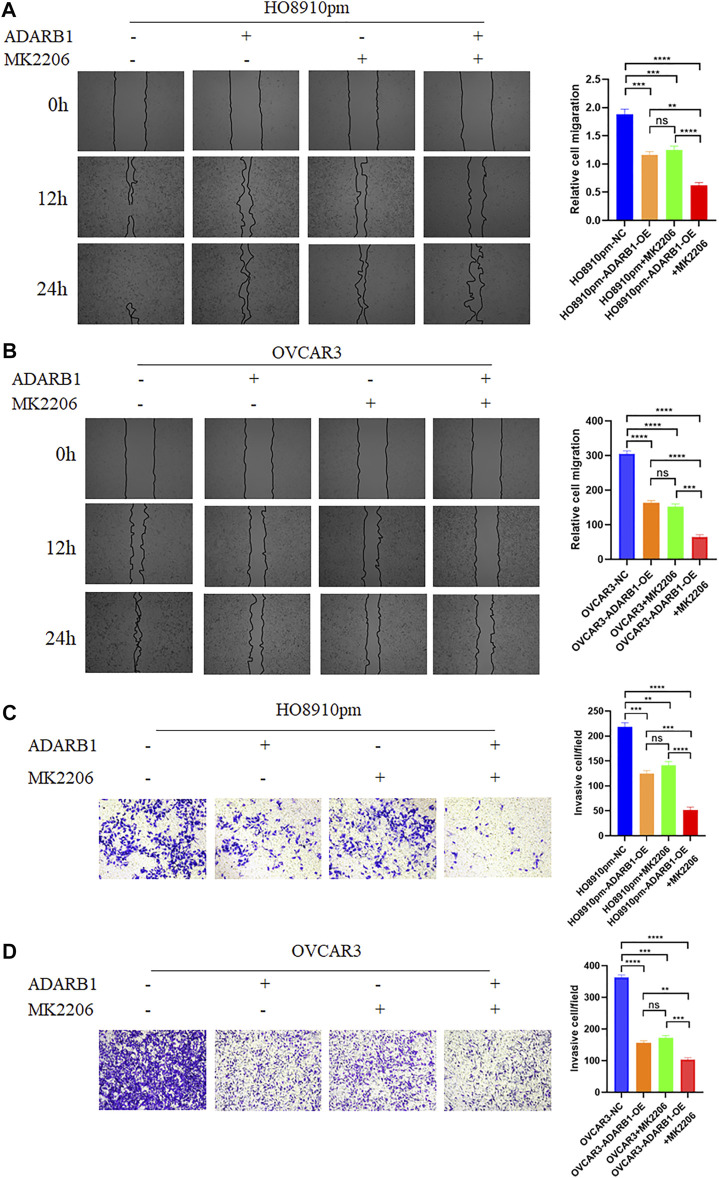
ADARB1 inhibits OC cell Metastasis and invasion by regulating AKT pathway. **(A,B)** The wound healing assays were performed after HO8910pm and OVCAR3 cells treated with ADARB1-OE and/or AKT inhibitors MK2206. **(C,D)** The transwell assay were performed after HO8910pm and OVCAR3 cells treated with ADARB1-OE and/or AKT inhibitors MK2206.

The PI3K-AKT pathway is a well-known tumor signaling pathway that is involved in the development of various tumors, including glioma (Dai et al., 2017), ovarian cancer (Wu et al., 2021), etc. Moreover, phosphorylation activation of AKT signaling can promote the malignant progression of ovarian cancer (Samartzis et al., 2020; Zhai et al., 2020). We found that the phosphorylation levels of AKT (p-AKT) in tumor tissues was higher than that in adjacent tissues (Supplementary Figure S1). And ADARB1-OE can effectively inhibit p-AKT without affecting the total AKT level. Then, we investigated whether the effect of ADARB1 on OC cell proliferation and metastasis was associated with AKT activity. Western blot analysis showed that p-AKT was obviously decreased when OC cells were overexpressed ADARB1 or treated with MK2206, a specific inhibitor of AKT. The inhibitory effect on p-AKT levels was further increased by combinational treatment (Figure 5A). We performed the MTT assay and colony formation assay and observed that OC cells treated with both MK2206 and ADARB1-OE had a significantly suppression effect of cell proliferation compared with the control group and either of the individual treatment groups (Figures 5B–D). Moreover, we examined cell migration and invasion capacity of HO8910pm and OVCAR3 cells under ADARB1-OE and MK2206 treatment. Compared to the control group, OC cells showed a significantly weaker migration and invasion ability when treated with MK2206 or ADARB1-OE (Figures 6A–D). The above results indicated that anti-cancer activity by ADARB1 overexpression might be due to the inhibition of AKT signaling.

### Functional Enrichment Analysis of ADARB1-Associated Coexpression Genes

**FIGURE 7:**
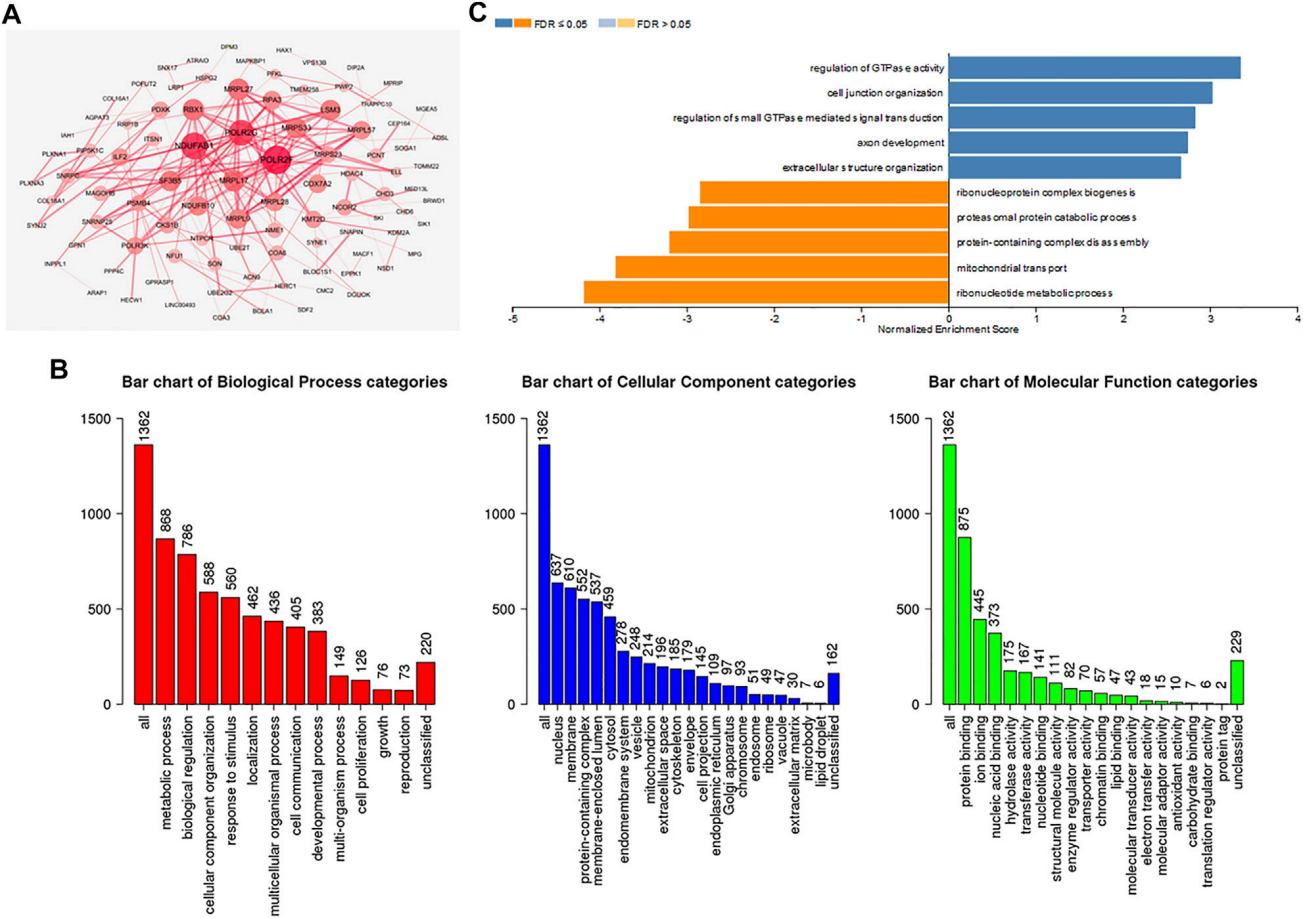
Functional enrichment analysis of ADARB1-associated coexpressed genes in OC. **(A)** A protein-protein interaction network of ADARB1-associated coexpressed genes drawn by the STRING and Cytoscape software. **(B)** GO analysis of ADARB1 associated coexpressed genes were identified by Webgestalt database. **(C)** The KEGG pathways of ADARB1-associated coexpressed genes were identified by Webgestalt database.

To further investigate the biological functions of ADARB1, functional enrichment analysis was performed using ADARB1 related coexpressed genes. By using the cBioPortal database, we screened 1399 ADARB1-associated coexpressed genes with the criteria of *p* value ≤ 0.05 and |Spearman’s correlation|≥0.3 (Supplementary Table S2). Next, a PPI network was performed using STRING and Cytoscape software (Figure 7A). Meanwhile, the GO annotation (Figure 7B) and KEGG pathway (Figure 7C) were further analyzed using WebGestalt database. Biological processes indicated that these coexpressed genes are mainly involved in metabolic processes and biological regulation. For cell component, these coexpressed genes were mainly located on the nucleus and membrane. In the aspect of molecular function, the coexpressed genes were primarily enriched in protein binding and ion binding. The KEGG pathway demonstrated that these coexpressed genes were mainly related to the ribonucleotide metabolic process and mitochondrial transport.

### Regulation of Immune Molecules by ADARB1

**FIGURE 8:**
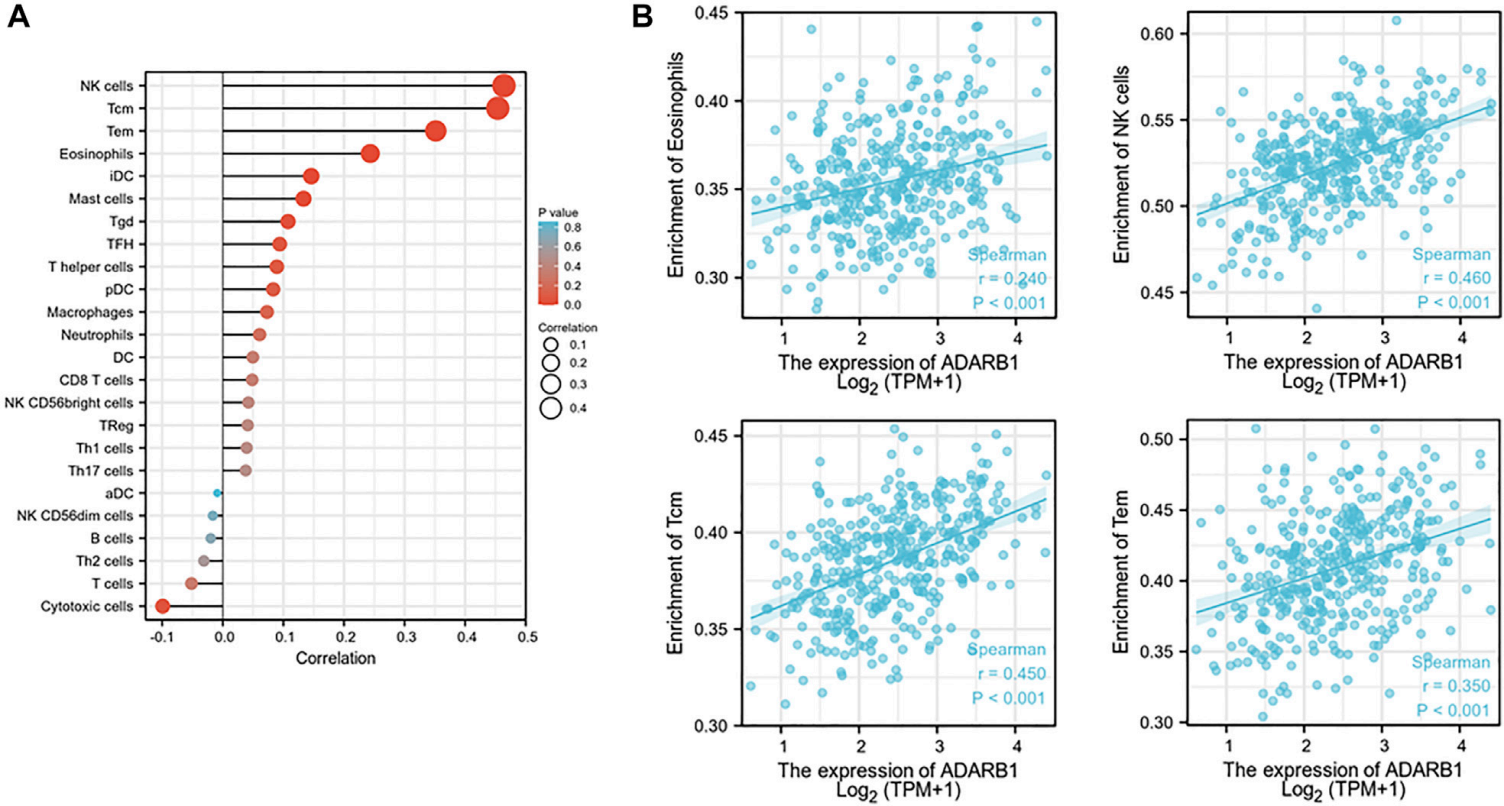
Correlation of ADARB1 expression with tumor-infiltrating lymphocytes in OC. **(A)** The correlation between ADARB1 expression and tumor-infiltrating lymphocytes. **(B)** The top four tumor-infiltrating lymphocytes showing the most significant correlations with ADARB1 expression. Supplementary Figure S1. Immunohistochemistry was used to examine the ADARB1,p-AKT, CXCL12 and KDR expression in ovarian cancer tissue and paired adjacent tissue (magnification 200×).

Increasing evidence demonstrated that ADAR family members play important roles in immune regulation (Heraud-Farlow and Walkley, 2020; Yanai et al., 2020). Therefore, from TISIDB database, we investigated the relationship between ADARB1 expression and immune infiltration. As shown in Figure 8A, the expression levels of ADARB1 positively correlated with several immune cells, such as natural killer cell (NK cells), central Memory T cell (Tcm), T effector memory (Tem) and eosinophils. Figure 8B further confirmed the positive correlation between ADARB1 expression and NK cells (Spearman r = 0.546), Tcm (Spearman r = 0.45), Tem (Spearman r = 0.35) and eosinophils (Spearman r = 0.24). Similarly, ADARB1 was positively correlated with eosinophil and NK cells by using the TISIDB database (Supplementary Figure S2). Moreover, we analyzed the associations between ADARB1 expression and the immunomodulators and chemokines. Supplementary Figure S3 showed the positive correlations between ADARB1 expression and several immunomodulators, including CD160 (Spearman r = 0.211), CSF1R (Spearman r = 0.231), KDR (Spearman r = 0.3) and TGFBR1 (Spearman r = 0.208). Supplementary Figures S4A,B showed the positive correlations between ADARB1 expression and several chemokines, including CCL14 (Spearman r = 0.153), CCL21 (Spearman r = 0.136), CXCL12 (Spearman r = 0.224) and CXCL14 (Spearman r = 0.18), while the ADARB1 expression levels were negative correlation with CCL13 (Spearman r = −0.13), CXCL8 (Spearman r = −0.143), CXCL13 (Spearman r = −0.129) and XCL1 (Spearman r = −0.194). In addition, we found the low level of ADARB1, KDR and CXCL2 in tumor tissues compared with the adjacent tissues by immunohistochemistry, which indicated that the expression of ADARB1 was positively correlated with the expression of KDR and CXCL12 (Supplementary Figure S1). We hypothesized that ADARB1 might have a significant effect on immune regulation in OC since it is obviously associated with various types of tumor-infiltrating lymphocytes, immunomodulators, and chemokines in OC.

## Discussion

This is the first study to investigate the expression and function of ADARB1 in OC and its association with clinical features from the perspective of bioinformatics. The results confirmed that the expression of ADARB1 was significantly reduced in OC tissues and cell lines. ADARB1 might display an anti-cancer role through inhibiting AKT phosphorylation. Moreover, patients with low expression of ADARB1 had shorter OS and PFS from DRUGSURV database and Kaplan-Meier Plotter database. However, we found the inconsistent prognostic data by using the Human Protein Altas database. These conflicting data may be due to different patients’ characteristics, such as age and race, in different databases. Thus, more OC patients from different regions will be needed for further confirmation the roles of ADARB1 in the future.

To date, ADAR has been widely found in all multicellular animal species. The mammalian genome has five genes encoding ADAR protein. Among these, ADAR1 and ADARB1 are active deaminases, ADAR3 has no known editing activity, and two other closely related testicle-specific ADAD1 and ADAD2 proteins lack the key catalytic residues (Goncharov et al., 2019). Recently, studies have demonstrated the key roles of ADARB1 in cancer development (Galeano et al., 2012; Goncharov et al., 2019). For example, hepatoma carcinoma patients with down-regulated ADARB1 expression have a poor prognosis (Chan et al., 2014). RNA editing of SLC22A3 regulated by ADARB1 promotes tumor invasion and metastasis in early familial esophageal carcinoma (Fu et al., 2017). In addition, ADARB1-OE promotes cell growth, motility, and invasion of malignant pleural mesothelioma cells independent of its RNA-editing activity (Sakata et al., 2020). However, the functional roles ADARB1 in OC pathology has not been investigated. In the present study, we demonstrated that ADARB1 expression was significantly down-regulated in OC tissues and cells, and might act as a tumor suppressor gene.

Alterations in AKT kinase activity are associated with a variety of pathologies. Increased AKT1 activity is associated with a variety of cancers, including OC (Gu et al., 2020; Samartzis et al., 2020; Zhai et al., 2020). Previous study has demonstrated that changes in AKT-dependent phosphorylation of ADARB1 have the potential to affect cellular programming (Piazzi et al., 2020). Similarly, the results in our study indicated that ADARB1-OE significantly inhibited tumor proliferation and metastasis through down-regulating AKT phosphorylation.

In addition, GO and KEGG pathway analysis indicated that genes coexpressed with ADARB1 were mainly enriched in ribonucleotide metabolic process, which suggested that ADARB1 might also be involved in OC progression by regulating these pathways. The metabolism was thought to sustained cancer cell malignant behaviors, including proliferation and metastasis, and anti-tumor immune function (Vaupel et al., 2019; Bergers and Fendt, 2021). To date, increasingly anti-tumor metabolism targets have been discovered, and have been used clinically for certain cancers (Luengo et al., 2017; Vander Heiden and DeBerardinis, 2017). Therefore, ADARB1 may be a useful molecular target for OC therapeutics.

In recent years, immunotherapy has become the focus of tumor therapy (Huang et al., 2020; Yan et al., 2020). T cell-rich tumor patients have longer PFS and OS (Zhang et al., 2003), while immune avoidance-associated mechanisms are associated with low survival (Kandalaft et al., 2009). All this evidence suggested that OC patients may benefit from immunotherapy (Ghisoni et al., 2019). In this study, TISIDB database was used to analyze the correlation between ADARB1 and immune system, and the results showed that ADARB1 had most significant correlation with several tumor-infiltrating lymphocytes (NK cells, Tcm, Tem, and eosinophils), immunomodulators (KDR, CSF1R, CD160 and TGFBR1), and chemokines (CXCL12, CCL14, CXCL14 and CCL21). ADARB1 knockout induces an antiviral immune response, leading to the suppression of infection (Yanai et al., 2020). Although these results suggested that ADARB1 may be related to tumor immunity, clinical research is needed for further study.

In summary, our study illustrated that low expression of ADARB1 is associated with poor prognosis in OC, suggesting that ADARB1 may be an anti-oncogene and could serve as a promising biomarker in the tumorigenesis of OC. Moreover, ADARB1 was found to be involved in AKT-mediated malignant biological properties of OC cells. In addition, ADARB1 expression is related to tumor-infiltrating lymphocytes and immunomodulators. Therefore, our findings suggested that ADARB1 likely plays a pivotal role in immune cell infiltration and could serve as a promising biomarker for prognosis in OC patients.

## Data Availability

The original contributions presented in the study are included in the article/Supplementary Material, further inquiries can be directed to the corresponding author.
