# Supplementary figures and images for: Downregulated ADARB1 Facilitates Cell Proliferation, Invasion and has Effect on the Immune Regulation in Ovarian Cancer
Source: Front Bioeng Biotechnol. 2021 Dec 23;9:792911. doi: 10.3389/fbioe.2021.792911 (PMC8733684; doi:10.3389/fbioe.2021.792911)

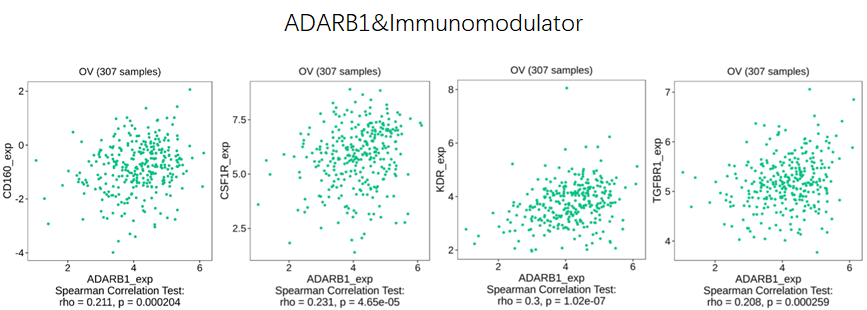

Supplement: Supplementary file 2 [file Image3.TIF]

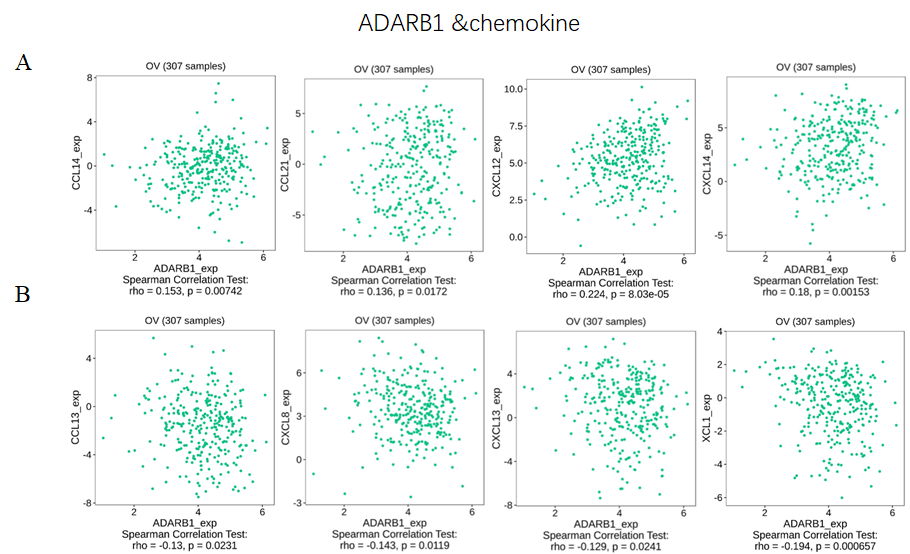

Supplement: Supplementary file 3 [file Image4.TIF]

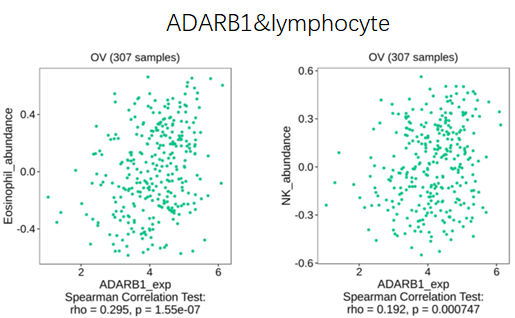

Supplement: Supplementary file 4 [file Image2.TIF]

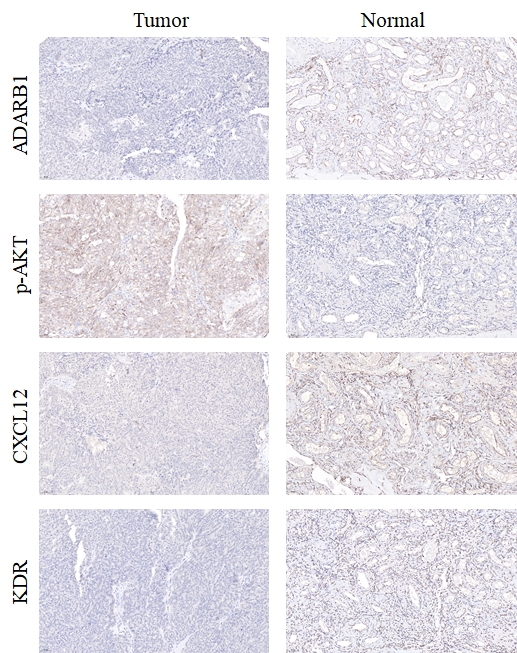

Supplement: Supplementary file 5 [file Image1.TIF]
